# No Effects of Bilateral tDCS over Inferior Frontal Gyrus on Response Inhibition and Aggression

**DOI:** 10.1371/journal.pone.0132170

**Published:** 2015-07-10

**Authors:** Franziska Dambacher, Teresa Schuhmann, Jill Lobbestael, Arnoud Arntz, Suzanne Brugman, Alexander T. Sack

**Affiliations:** 1 Department of Cognitive Neuroscience, Maastricht University, Maastricht, The Netherlands; 2 Department of Clinical Psychological Science, Maastricht University, Maastricht, The Netherlands; 3 Maastricht Brain Imaging Center, Maastricht, The Netherlands; 4 Department of Clinical Psychology, University of Amsterdam, Amsterdam, The Netherlands; UCLA, UNITED STATES

## Abstract

Response inhibition is defined as the capacity to adequately withdraw pre-planned responses. It has been shown that individuals with deficits in inhibiting pre-planned responses tend to display more aggressive behaviour. The prefrontal cortex is involved in both, response inhibition and aggression. While response inhibition is mostly associated with predominantly right prefrontal activity, the neural components underlying aggression seem to be left-lateralized. These differences in hemispheric dominance are conceptualized in cortical asymmetry theories on motivational direction, which assign avoidance motivation (relevant to inhibit responses) to the right and approach motivation (relevant for aggressive actions) to the left prefrontal cortex. The current study aimed to directly address the inverse relationship between response inhibition and aggression by assessing them within one experiment. Sixty-nine healthy participants underwent bilateral transcranial Direct Current Stimulation (tDCS) to the inferior frontal cortex. In one group we induced right-hemispheric fronto-cortical dominance by means of a combined right prefrontal anodal and left prefrontal cathodal tDCS montage. In a second group we induced left-hemispheric fronto-cortical dominance by means of a combined left prefrontal anodal and right prefrontal cathodal tDCS montage. A control group received sham stimulation. Response inhibition was assessed with a go/no-go task (GNGT) and aggression with the Taylor Aggression Paradigm (TAP). We revealed that participants with poorer performance in the GNGT displayed more aggression during the TAP. No effects of bilateral prefrontal tDCS on either response inhibition or aggression were observed. This is at odds with previous brain stimulation studies applying unilateral protocols. Our results failed to provide evidence in support of the prefrontal cortical asymmetry model in the domain of response inhibition and aggression. The absence of tDCS effects might also indicate that the methodological approach of shifting cortical asymmetry by means of bilateral tDCS protocols has failed.

## Introduction

Response inhibition is defined as the cognitive ability to withhold automatic or pre-planned reactions [1]. It comprises various sub-components including action restraint which refers to the withdrawal of an action prior to its initiation [2]. Action restraint is classically measured by go/no-go paradigms in which participants have to respond to a frequent go stimulus, while they have to restrain their response to an infrequent no-go stimulus.

Aggression is understood as behaviour that aims to intentionally harm another being verbally, physically, or psychologically [3]. It is usually categorized into sub-types, namely proactive and reactive aggression. While proactive aggression refers to using aggression in an instrumental goal-oriented way, reactive aggression refers to aggressive actions in response to preceding provocation [4] [5]. Aggression should not only be assessed by means of self-report, but also by means of controlled behavioural paradigms such as the Taylor Aggression Paradigm (TAP) [6]. The TAP is set up as a reaction time game in which two or more opponents interact and are enabled to administer aversive feedback of variable intensity to each other. In the paradigm, behavioural aggression is measured through the intensity of feedback a participant choses for the opponent.

Response inhibition has repeatedly been linked with aggression [7]. For example, research has focused on the underlying neural correlates of response inhibition, self-reported impulsivity, and aggression. Pawliczek and colleagues (2013) demonstrated that highly aggressive individuals display inhibition deficits in an emotional stop signal task and lower inhibition related brain activity in pre-supplementary motor area and primary motor cortex. More recently, a substantial overlap of neural networks involved in failed response inhibition and behavioural aggression within anterior insula and various sub-cortical brain regions was demonstrated [8]. Horn and colleagues [9] showed that impulsive individuals recruited more activity in the right orbitofrontal cortex to maintain inhibitory capacities in a go/no-go task (GNGT) compared to non-impulsive individuals. Furthermore, GNGT-inhibition related activity in the right dorsolateral prefrontal cortex was shown to negatively correlate with impulsiveness [10].

Despite the described relationship between response inhibition and aggression, neuroscientific research has mostly examined those concepts in isolation. Response inhibition is mainly associated with activity in the right prefrontal cortex [11] [12], while anger and aggression are mainly associated with activity in the left prefrontal cortex [10] [13] [14]. Studies employing non-invasive brain stimulation such as transcranial Direct Current Stimulation (tDCS) and Transcranial Magnetic Stimulation (TMS) suggest that response inhibition can be altered by changing activity in the right prefrontal cortex: Jacobson and colleagues [15] showed that enhancing activity in right inferior frontal gyrus by means of unilateral anodal tDCS improved inhibition in a stop signal task. In a similar vein, disrupting right inferior frontal cortex with TMS [16–19] impaired successful inhibition in various response inhibition paradigms. Inhibiting right dorsolateral prefrontal cortex by means of cathodal tDCS [20] led to comparable results.

When studying aggression, non-invasive brain stimulation findings suggest that aggression and its cognitive predecessors can be increased by shifting the fronto-cortical balance towards the left hemisphere and decreased when shifting the balance to the right. D’Alfonso and colleagues [21] demonstrated that by disrupting the right prefrontal cortex with TMS an attentional bias towards angry faces was induced, while the disruption of left prefrontal cortex had an opposite effect. When enhancing the left prefrontal cortex with tDCS, aggressive behaviour in provocative situations was increased [22]. Opposite effects (decrease of proactive aggression in males) were observed, when unilaterally enhancing the right dorsolateral prefrontal cortex [23].

In summary, non-invasive brain stimulation studies showed that shifting the fronto-cortical dominance towards the right hemisphere (by enhancing the right and/or disrupting the left prefrontal cortex) increases inhibitory capacity and decreases aggression, while shifting it to the left (by enhancing the left and/or disrupting the right prefrontal cortex) had the opposite effect. These results are in line with the theoretical framework on fronto-cortical asymmetry and motivational states proposed by Harmon-Jones and colleagues [24–26]. It states that avoidance motivation is associated with right-hemispheric fronto-cortical brain activity, while approach motivation is associated with left- hemispheric fronto cortical activity. Although the fact that action restraint (related to avoidance motivation) and aggression (related to approach motivation) seem inversely related on behavioural and neural level fits with this framework, no brain stimulation study aimed to directly address this relationship by assessing both concepts within one experiment and by applying two opposing bilateral brain stimulation protocols to induce left and right fronto-cortical dominance, respectively.

In the current study we applied bilateral tDCS, inducing either right or left fronto-cortical dominance, or sham stimulation, while participants were performing a response inhibition task (GNGT) and a behavioural aggression paradigm (Taylor Aggression Paradigm). The inferior frontal cortex was targeted, based on previous neuroimaging work [10]. We assumed that response inhibition and aggression were inversely related on both behavioural and neural level. We hypothesized that the induction of right-hemispheric fronto-cortical dominance by means of a combined right prefrontal anodal and left prefrontal cathodal tDCS montage would enhance the ability to inhibit motor responses and at the same time reduce aggressive behaviour. The induction of left-hemispheric fronto-cortical dominance by means of a combined left prefrontal anodal and right prefrontal cathodal tDCS montage on the other hand was expected to reduce the ability to inhibit motor responses and at the same time increase aggressive behaviour.

## Methods and Materials

### Participants

Sixty-nine healthy volunteers participated in the study. Data of one participant was incomplete due to technical problems and had to be excluded from the analysis. Another four participants were excluded from the analysis, as they doubted the interaction with a real human opponent during the TAP (see below). Sixty-four participants (mean age in years = 21.89; SD = 3.26) were, thus, included in the analysis. Participants were randomly assigned to one of three experimental groups (between-subject design): One group received anodal stimulation over right and simultaneously cathodal stimulation over left inferior frontal cortex (induction of right-hemispheric dominance; male n = 11 female n = 11). The second group received anodal stimulation over left and simultaneously cathodal stimulation over right inferior frontal cortex (induction of left-hemispheric dominance; male n = 14 female n = 8). A third group received sham stimulation (male n = 14 female n = 6).Participants did not have a history of neurological or psychiatric disorders and gave their written informed consent prior to participation.

### Paradigms and tools

#### GNGT

To measure response inhibition, a standard go/no-go motor response task was employed [18]. Participants were instructed to respond as fast and accurately as possible to a frequent go stimulus via button press, while retraining their response to a rare no-go stimulus. Go as well as no-go stimuli were presented for 100 msec. Inter trial intervals were randomly varied (650, 750, 850, 950, or 1050 msec) eliminating expectancy effects. The letters C and M were used as stimuli, as they lack any linguistic association with the concept of “stopping” in the languages spoken by the participants. Stimuli and fixation crosses were presented in white (RGB 255/255/255; Arial pt 24) on a grey background (RGB 125/125/125). For both the baseline and the actual experimental measurement, participants had to complete 5 blocks of 64 trials including 25% inhibition trials. Go and no-go trials were pseudo-randomized (one of four trials was an inhibition trial). This design led to a total of 320 trials (80 inhibition trials). After each block participants received feedback on their mean reaction times for go trials, their number of omission errors in go trials, and their percentage of commission errors in inhibition trials. Stimuli were presented using Presentation software (Neurobehavioural Systems, Inc., Albany, USA).

#### TAP

To measure actual aggressive behaviour, the Taylor Aggression Paradigm (TAP) [6] implemented in custom-made software was employed. The TAP is a paradigm that has demonstrated high construct, internal, discriminant as well as external validity [27–30]. In our case it was framed as a competitive reaction time game in which participants had to respond to a target stimulus as fast as possible by button press with the right index finger. Two participants were simultaneously invited to the laboratory. Each participant was told to play against another participant of the same gender sitting in the next room. The amount of win and lose trials were preprogrammed in the same order for every participant. Participants were made to believe that the winner of a trial could administer an aversive noise to the opponent and that this noise could influence the performance of the opponent on the next trial. In the beginning of each trial, the participant was asked to choose the duration and volume of this noise blast by moving a slider on a scale from 0 to 10 (duration: 0 to 5 seconds; volume: 0 to 100 dB). At the end of each trial, participants were informed about whether they had won or lost the trial. At the same time they could see which feedback the opponent had chosen for the trial. In the case that the participant had lost, she / he was presented with this feedback through headphones. By summing and averaging the behaviour (given intensity & duration) across all trials, a total aggression score was calculated. The behaviour (given intensity & duration) across the first seven unprovoked trials (in which the opponent never administered a noise) was summed up and averaged to calculate a *proactive aggression score*. By summing and averaging the behaviour (given intensity & duration) from the eighth trial onwards (provoked trials), a *reactive aggression score* was calculated.

#### Questionnaires

The Reactive-Proactive-Aggression Questionnaire (RPQ) was used to measure self-reported trait aggression [5]. Participants rated their opponents regarding sympathy, competence, friendliness, and reaction time speed on a 7-point likert scale at the beginning and the end of the experiment. In order to check, whether the actual behaviour (administration of feedback noise) and not merely the perception of the feedback received by the opponent was modulated by tDCS, participants rated on a 7-point likert scale how annoying 4 exemplary feedbacks would have been for them, if they would have received them during the game (‘volume 0 / duration 0’, ‘volume 4 / duration 2’, ‘volume 10 / duration 10’, ‘volume 2 / duration 6’). During this rating the brain stimulation was still active.

### Non-invasive brain stimulation

Participants were split into three groups (between-subject design) and randomly assigned to one of three tDCS conditions: induction of right-hemispheric fronto-cortical dominance, induction of left-hemispheric fronto-cortical dominance, or sham stimulation.

**Fig 1:**
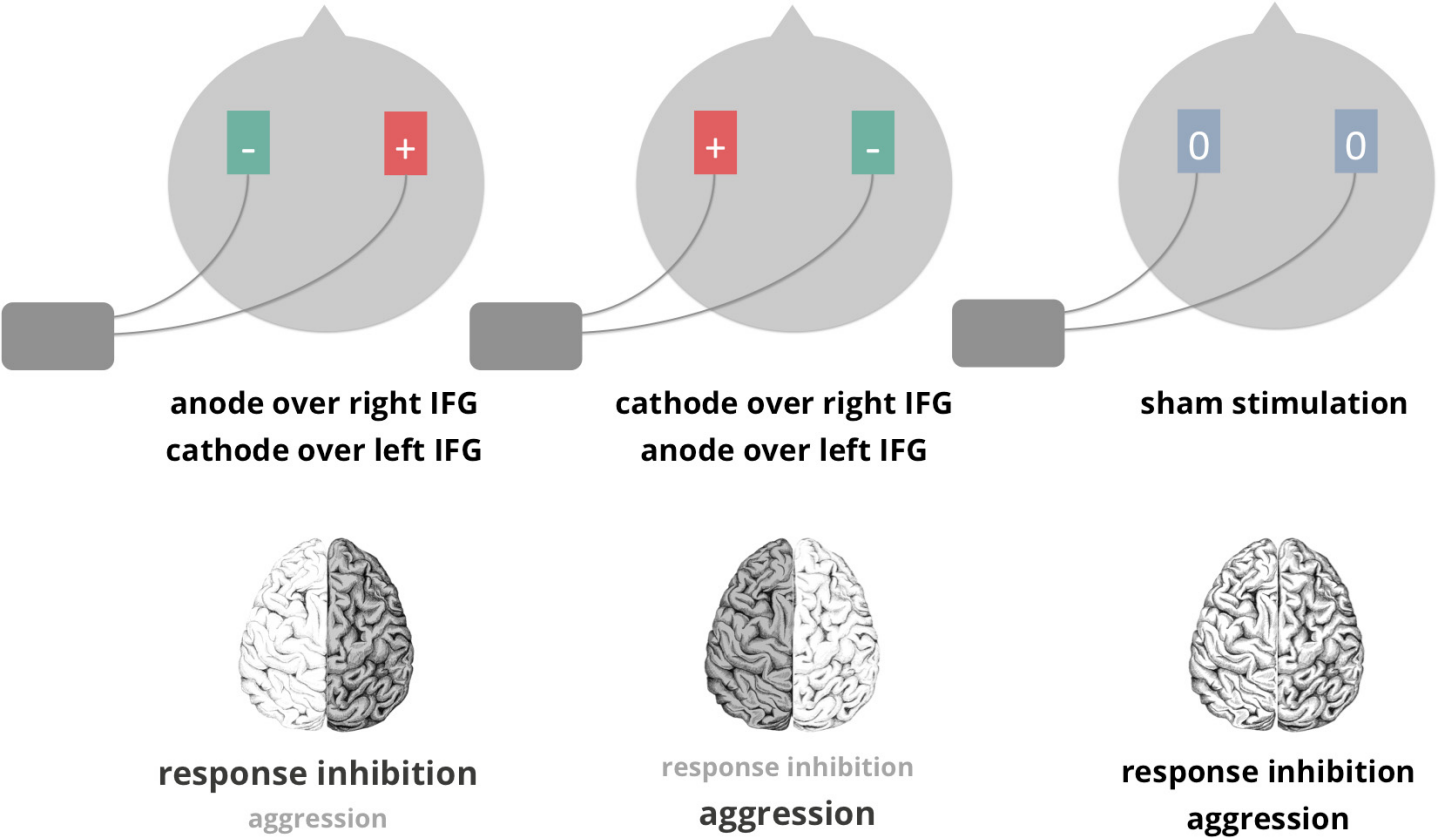
Experimental design & sketched hypotheses. IFG: inferior frontal gyrus.

To induce right-hemispheric fronto-cortical dominance, the anode was positioned over right inferior frontal cortex (F8), while the cathode was positioned over left inferior frontal cortex (F7). Induction of left-hemispheric fronto-cortical dominance was achieved by positioning the anode over left inferior frontal cortex (F7) and the cathode over right inferior frontal cortex (F8). F7 and F8 were localized according to the international 10–20 system. These stimulation sites were based on existing imaging work allocating the main overlap of neural networks involved in response inhibition and aggression in inferior frontal regions [10] [12]. A bilateral protocol was chosen based on the assumption that enhancing the excitability of one, while decreasing the excitability of the other hemisphere, would shift the fronto-cortical balance to a greater extent than a unilateral protocol. A DC-stimulator plus and 5x7cm standard electrodes (neuroConn, Ilmenau, Germany) were employed. Electrodes were fixated using conductive EEG gel (Ten20 conductive Neurodiagnostic electrode paste, WEAVER and company, Aurora CO, USA). Participants received stimulation at intensity of 1.5mA. This intensity was experienced as comfortable by the participants and was in the middle of two intensities most frequently reported in the literature (1mA vs. 2mA). Current was applied for 21.75 minutes, including ramping up and down phases of 20s. Stimulation parameters are visualized in Fig 1.

When administering sham tDCS the electrodes were also positioned over F7 and F8, but the stimulation was switched off immediately after the ramping phase. Participants thus experienced a light tingling sensation in the sham condition. Participants could not differentiate whether they had been assigned to the real or the sham tDCS condition. When rating how certain they were about which type of stimulation they received (from 1 “100% sham” to 7 “100% real”; with 4 “I don’t know”), no differences between the real and sham stimulation groups were identified (*right-hemispheric fronto-cortical dominance*: MEAN = 4.73; *left-hemispheric fronto-cortical dominance*: MEAN = 4.67; *sham stimulation*: MEAN = 4.75; ANOVA: F(2,61) = .017 p = .984).

Participants reported no side effects of tDCS stimulation except a tingling or burning sensation at the beginning and the end of stimulation; for few participants slightly reddened skin was observed after stimulation on locations where the electrodes had been placed.

### Experimental procedure

Participants were told that they took part in a study investigating the effects of human feedback compared to computerized feedback in reaction time performance. In each experimental session two participants of the same gender took part simultaneously. Participants were seated in two different adjacent laboratory rooms. Following the montage of the tDCS setup, participants received instructions and completed a baseline measurement of the GNGT. During brain stimulation (the experimental manipulation) participants performed the GNGT and the TAP in counterbalanced order. Subsequently, participants had to fill in the questionnaires. Immediately after completion of the experiment, participants had an exit interview checking whether they were fully deceived by the experimental setup. Participants were provided with a written debriefing upon completion of measurements.

### Ethical statement

The study was approved by the local Ethical Committee of the Faculty of Psychology and Neuroscience at Maastricht University.

### Statistical analysis

The effects of brain stimulation on response inhibition were examined with a 3x2 multivariate analysis of variance (MANOVA; conditions x gender; with mean reaction time on go trials, misses, and false alarms as dependent variables; all corrected for baseline via differential scores). The effects of brain stimulation on aggression were examined with a 3x2 multivariate analysis of variance (MANOVA; conditions x gender; with total aggression, proactive aggression, and reactive aggression as dependent variables); trait aggression (RPQ) was included as a covariate. As post-hoc test (investigating specific differences) paired-sample t-tests were performed. Relationships between variables were investigated via Pearson product-moment correlation coefficients. Post hoc analyses were conducted with G-power software [31] to ensure sufficient statistical power.

## Results

Raw data can be found in the supplementary material (S1 Raw Data).

In the GNGT, participants in the control group (receiving sham stimulation) became faster and committed more commission errors (false alarms) in the experimental measurement compared to the baseline measurement (*reaction time*: MEANpre = 292.21 MEANpost = 271.83 t(19) = 5.686 p < .001; *false alarms*: MEANpre = 22.20 MEANpost = 30.00 t(19) = -3.765 p = .001). Therefore, in all three groups further analyses for the GNGT were computed on differential scores (GNGT variables during brain stimulation minus GNGT variables baseline). Inhibitory capacity in the GNGT also correlated with all types of aggression in the TAP. The more false alarms participants committed, the more aggression they displayed (TOTAL AGGRESSION r = .550 p = .012 / PROACTIVE AGGRESSION r = .452 p = .046 / REACTIVE AGGRESSION r = .474 p = .035). No such relation could be observed within the experimental groups (receiving real stimulation).

### Effects of brain stimulation

Means and standard deviations of all dependent variables are depicted in Table 1. Analyzing the effects of brain stimulation on response inhibition, a 3x2 MANOVA (conditions x gender; with mean reaction time on go trials, misses, and false alarms as dependent variables; all corrected for baseline) revealed no significant main effects (MEAN REACTION TIME *condition*: F(2,61) = 2.293 p = .100; *gender*: F(1,62) = 2.100 p = .153 / MISSES *condition*: F(2,61) = 1.118 p = .334; *gender*: F(1,62) = 1.949 p = .168 / FALSE ALARMS *condition*: F(2,61) = 2.193 p = .121; *gender*: F(1,62) = .341 p = .562). No interaction effects were observed (MEAN REACTION TIME condition*gender: F = .064 p = .938 / MISSES condition*gender: F = 2.550 p = .087 / FALSE ALARMS condition*gender: F = .032 p = .969). A post-hoc power analysis was performed for the analysis reported in this paragraph. It revealed an achieved power of .95 for both main effects and the interaction (assuming alpha = .05, 1-beta = .95, based on Pillai’s V per effect; calculated with G-Power).

**Table 1:** Means and standard deviations. Reaction times, misses, and false alarms are represented as differential values (minus baseline performance). Agg: aggression.

	induction of right-hemispheric dominance				induction of left-hemispheric dominance				sham stimulation			
	*male* n = 11		*female* n = 11		*male* n = 14		*female* n = 8		*male* n = 14		*female* n = 6	
	M	SD	M	SD	M	SD	M	SD	M	SD	M	SD
*reaction time*	-22.47	12.50	-30.84	21.88	-9.49	23.61	-18.20	19.70	-19.00	16.57	-23.61	15.64
*misses*	-.27	12.50	-10.55	7.78	-4.14	8.49	-1.38	10.36	0.50	9.20	-2.33	6.34
*false alarms*	4.55	5.26	6.36	8.04	2.29	6.53	2.88	8.98	7.43	10.65	8.67	5.50
*total agg*	5.10	1.31	3.85	1.05	4.26	1.73	2.98	1.52	4.45	1.18	4.73	.90
*proactive agg*	4.19	1.76	2.39	1.32	3.52	1.62	1.82	.95	3.37	1.66	3.35	.75
*reactive agg*	5.33	1.26	4.22	1.05	4.45	1.89	3.27	1.86	4.73	1.33	5.07	1.06

Analyzing the effects of brain stimulation on aggression, a 3x2 MANOVA (conditions x gender; with total aggression, proactive aggression and reactive aggression as dependent variable and trait aggression as covariate) revealed a significant gender difference in proactive aggression with males displaying more proactive aggression than females (PROACTIVE AGGRESSION *gender*: F(1,62) = 7.142 p = .010). No other significant main effects were revealed (TOTAL AGGRESSION *condition*: F(2,61) = 1.906 p = .159; *gender*: F(1,62) = 3.459 p = .068 / PROACTIVE AGGRESSION *condition*: F(2,61) = 1.060 p = .354 / REACTIVE AGGRESSION *condition*: F(2,61) = 1.759 p = .182; *gender*: F(1,62) = 2.07 p = .155). No interaction effects were observed (TOTAL AGGRESSION condition*gender: F = .960 p = .389 / PROACTIVE AGGRESSION condition*gender: F = 1.575 p = .216 / REACTIVE AGGRESSION condition*gender: F = .645 p = .529). A post-hoc power analysis was performed for the analysis reported in this paragraph. It revealed an achieved power of .95 for both main effects and the interaction (assuming alpha = .05, 1-beta = .95, based on Pillai’s V per effect; calculated with G-Power).

### Control variables

We analyzed how participants perceived their opponent and the reaction time competition. Participants considered their opponent more competent, but less friendly after they had played the TAP (*competence pre/post*: MEANpre = 4.98 MEANpost = 5.50 / t(63) = -3.014 p = .004; *friendliness pre/post*: MEANpre = 5.67 MEANpost = 4.83 / t(63) = 5.294 p = .000; all items rated on a 7point Likert-Scale). No significant differences in the items *How much do you like your opponent* (*pre/post*: t(63) = .904 p = .369) and *How fast in his reactions will (was) your opponent be (pre/post*: t(63) = 1.263 p = .211) were observed. The higher in volume and duration the feedback was, the more annoying it was perceived (rated from 1 “not at all annoying” to 7 “extremely annoying”); this observation did not differ with respect to the brain stimulation conditions (*right-hemispheric fronto-cortical dominance*: feedback volume 0 & duration 0 MEAN = 1.50 / feedback volume 4 & duration 1 MEAN = 2.59 / feedback volume 10 & duration 5 MEAN = 6.59 / feedback volume 2 & duration 3 MEAN = 2.64; *left-hemispheric fronto-cortical dominance*: feedback volume 0 & duration 0 MEAN = 1.64 / feedback volume 4 & duration 1 MEAN = 2.68 / feedback volume 10 & duration 5 MEAN = 6.36 / feedback volume 2 & duration 3 MEAN = 2.68; *sham stimulation*: feedback volume 0 & duration 0 MEAN = 1.30 / feedback volume 4 & duration 1 MEAN = 2.70 / feedback volume 10 & duration 5 MEAN = 6.30 / feedback volume 2 & duration 3 MEAN = 2.65).

## Discussion

We investigated whether shifting fronto-cortical balance with bilateral tDCS affects response inhibition and aggression. Assuming that response inhibition and aggression are inversely correlated on behavioural and neural level, we expected that the induction of right-hemispheric fronto-cortical dominance by means of a combined right prefrontal anodal and left prefrontal cathodal tDCS montage would enhance the ability to inhibit motor responses and at the same time reduce aggressive behaviour. The induction of left-hemispheric fronto-cortical dominance by means of a combined left prefrontal anodal and right prefrontal cathodal tDCS montage on the other hand was expected to reduce the ability to inhibit motor responses and at the same time increase aggressive behaviour. We failed to reveal a behavioural effect of either tDCS condition on response inhibition and aggression and could not provide empirical support for these hypotheses.

Our aggression measure was effective: The interaction with an opponent during the reaction time game caused participants to evaluate their opponents as less friendly but more competent after having played the game as compared to the beginning of the experiment. This shows that the experimental situation was perceived as competitive and provocative. Furthermore, the louder and longer the received feedback noises were, the more annoying participants rated them. Both results indicate that the implementation of provocation in the TAP was successful. Over all conditions, males showed more proactive aggression than females. A strong gender effect in the context of the TAP has been shown before [23]. This is in line with a vast body of evidence supporting the notion that males tend to display more overt aggression than females especially in the domain of physical aggression as assessed by the TAP [32–35].

When examining the relation between response inhibition and aggression, an inverse correlation between the ability to inhibit pre-planned motor responses and all types of behavioural aggression (proactive, reactive, and total aggression) was observed in the control group (receiving sham stimulation). The more false alarms (commission errors) were committed by a given participant in the GNGT, the more aggression was displayed in the TAP. In other words: the worse people were in restraining motor responses when asked to do so in a response inhibition paradigm, the more aggressively they behaved towards their opponent after provocation within a social interaction paradigm. This is in line with previous work associating response inhibition deficits with impulsivity [8] [9] [36]. The current study, however, is the first to demonstrate a comparable relationship with respect to impulsive aggression employing an actual behavioural measurement instead of a mere self-report measure.

The described inverse correlation between response inhibition and aggression was only observed in the group receiving sham stimulation, but not in the experimental groups. Although not related to our a priori hypothesis and the framework of cortical asymmetry theories and motivational direction, this might hint towards the fact that non-invasive brain stimulation might affect the relationship between different behavioural constructs rather than the behaviour per se. Another explanation might be that tDCS could have affected the speed-accuracy trade off rather than absolute response inhibition performance. Unfortunately, we were not able to test this directly due to the small number of participants per group.

Our null results are at odds with previous studies in which stimulation was applied unilaterally and led to a modification in response inhibition and / or aggression: Anodal stimulation applied unilaterally to the right inferior frontal cortex enhanced the ability to inhibit responses in a stop signal paradigm [15]. Applying cathodal stimulation unilaterally to the right dorsolateral prefrontal cortex was shown to impair response inhibition in a GNGT [20]. Finally, applying anodal stimulation unilaterally to the right dorsolateral prefrontal cortex was shown to reduce proactive aggression in males [23].

Although this study was designed based on sufficient statistical power (as revealed by post-hoc power analyses), the employed bilateral tDCS protocol had no effect on either response inhibition or aggression. This could mean on one hand that our tDCS findings fail to find evidence in favor of the hypothesized prefrontal cortical asymmetry in the domain of response inhibition and aggression. On the other hand, the current results, which were obtained based on a clear hypothesis and sound methodology, can also give indications on the (in)efficacy of the brain stimulation parameters and stimulation sites chosen:

However, it is not possible to draw clear conclusion in this case, as the ‘absence of evidence’ in brain stimulation research cannot simply be interpreted as ‘evidence of absence’ [37].

The stimulation could have been inefficient due to the bilateral montage. Based on a concrete hypothesis derived from the expected inverse relationship between response inhibition and aggression and their opposing prefrontal lateralization, we opted for a bilateral stimulation protocol positioning both electrodes symmetrically over both hemispheres. This is opposed to the usage of a unilateral (or non-symmetrical) protocol, for which the return electrode would be positioned, for instance, over the orbit or mastoid of the hemisphere contralateral to the target site. We assumed that the here employed setup of enhancing activity in one hemisphere, while at the same time inhibiting activity in the region symmetric to the target site in the contralateral hemisphere, would directly manipulate the prefrontal cortical asymmetry underlying response inhibition and aggression. However, as of yet, no empirical evidence exists that supports the general validity of such bilateral tDCS montages, neither on behavioural nor neurophysiological level [38] [39]. It thus remains speculative whether bilateral tDCS protocols as described in the current study in fact induce the intended shifts in cortical balance between hemispheres. It might very well be the case that such bilateral montages induce other neural effects, e.g. homeostatic plasticity [40] [41]. It has been shown–mainly in the motor domain–, that neural activity tends to stabilize at a certain set point. When excitability in one region is compromised, other regions take over. The activation of a region due to task performance or training can counteract the brain stimulation induced inhibition of the region. Mechanisms of homeostatic plasticity that might be in place when applying bilateral tDCS protocols could account for our null results.

Our choice of target site might not have been optimal. Imaging work, on which we based our tDCS target regions, shows that the main overlap of neural networks involved in response inhibition and aggression is in inferior frontal cortex [10] [12]. However, previous studies demonstrating tDCS effects on response inhibition or aggression have positioned the electrodes superior to our target sites within dorsolateral prefrontal cortex [20] [22] [23].

Our tDCS intervention might have failed to reveal any effect, because we might have chosen sub-optimal stimulation parameters: Stimulation intensity might have been too low. In order to maximize participant comfort we chose to stimulate with 1.5mA. This intensity was rather low compared to tDCS studies which observed a brain stimulation related effect after stimulating with 2.0mA [22] [23]. It might have been preferable to use smaller electrodes to produce more focal current density in combination with a higher intensity to increase the efficacy of tDCS. Furthermore, bilateral offline protocols of 2mA have been applied successfully to the right dorsolateral prefrontal cortex before [42]. It might be that observing offline instead of online tDCS effects might have altered our results.

As no study has applied an online bilateral tDCS protocol specifically over inferior frontal cortex, the comparability of our study to previous findings is limited and we can only speculate on the reasons for the absence of a tDCS effect. Our current study in any of these cases indicates that caution is warranted when conceptualizing the manipulation of hemispheric asymmetry by means of bilateral tDCS montages.

## Supporting Information

S1 Raw Data In the supplementary material (S1) we provided our anonymized raw data. (ZIP)
